# Dual Roles of Prolactin and Vasoinhibin in Inflammatory Arthritis

**DOI:** 10.3389/fendo.2022.905756

**Published:** 2022-06-02

**Authors:** Carmen Clapp, Georgina Ortiz, Jose F. García-Rodrigo, María G. Ledesma-Colunga, Oscar F. Martínez-Díaz, Norma Adán, Gonzalo Martínez de la Escalera

**Affiliations:** Instituto de Neurobiología, Universidad Nacional Autónoma de México (UNAM), Campus UNAM-Juriquilla, Querétaro, Mexico

**Keywords:** rheumatoid arthritis, proinflammatory cytokines, joint inflammation, angiogenesis, synovial fibroblasts, endothelial cells, prolactin, vasoinhibin

## Abstract

The term inflammatory arthritis defines a family of diseases, including rheumatoid arthritis (RA), caused by an overactive immune system, and influenced by host aspects including sex, reproductive state, and stress. Prolactin (PRL) is a sexually dimorphic, reproductive, stress-related hormone long-linked to RA under the general assumption that it aggravates the disease. However, this conclusion remains controversial since PRL has both negative and positive outcomes in RA that may depend on the hormone circulating levels, synthesis by joint tissues, and complex interactions at the inflammatory milieu. The inflamed joint is rich in matrix metalloproteases that cleave PRL to vasoinhibin, a PRL fragment with proinflammatory effects and the ability to inhibit the hyperpermeability and growth of blood vessels. This review addresses this field with the idea that explanatory mechanisms lie within the PRL/vasoinhibin axis, an integrative framework influencing not only the levels of systemic and local PRL, but also the proteolytic conversion of PRL to vasoinhibin, as vasoinhibin itself has dual actions on joint inflammation. In this review, we discuss recent findings from mouse models suggesting the upregulation of endogenous vasoinhibin by the pro-inflammatory environment and showing dichotomous actions and signaling mechanisms of PRL and vasoinhibin on joint inflammation that are cell-specific and context-dependent. We hypothesize that these opposing actions work together to balance the inflammatory response and provide new insights for understanding the pathophysiology of RA and the development of new treatments.

## Introduction

Inflammatory arthritis is a collective name for a group of acute and chronic diseases driven by an overactive immune system that causes painful inflammation and stiffness of one or more articular joints. These diseases, broadly classified as non-autoimmune (sepsis arthritis and gout) and autoimmune (rheumatoid arthritis, juvenile idiopathic arthritis, spondyloarthritis, among others) are progressively debilitating if untreated. Rheumatoid arthritis (RA) is the most common chronic inflammatory arthritis affecting around 1% of the global population with a female to male ratio 3 to 1 (1). RA manifests as progressive synovial hyperplasia (pannus formation) and inflammation (synovitis) leading to polyarticular destruction. The etiology of RA is multifactorial, with genetic, environmental, and host-related factors driving early alterations of the innate and adaptive immune system that result in the recruitment of immune cells into the joints and subsequent chronic inflammation.

The close association between RA, sex, reproductive state, and stress have long-linked the sexually dimorphic, reproductive, stress-related hormone prolactin (PRL) to disease progression (2). However, the role of PRL in RA is more complex than anticipated. Clinical and pre-clinical studies have shown that PRL can be both pro-inflammatory and anti-inflammatory in a context-dependent manner. The detailed essentials of the association of PRL and RA are beyond the scope of this article and can be found in several reviews (2–5). Here, we briefly summarize the bases of the association between PRL and RA and focus on recent findings in arthritic rodents showing direct effects of PRL on joint tissues and the influence of the proteolytic conversion of PRL to vasoinhibin, a PRL fragment with dual actions on vascular and non-vascular cells of joint tissues that affect inflammatory reactions. Finally, we discuss how this information may be translated into novel therapeutic interventions.

## Prolactin and RA

The fact that RA is more frequent in women and disparity is greater at younger ages (6) encouraged investigating the female reproductive history. Early studies showed a higher risk of RA in nulliparous than parous women and a higher risk and worsening of RA in the postpartum period in association with breastfeeding (7). Because both risk factors (reduced fecundity and breastfeeding) associate with hyperprolactinemia, PRL was suggested as a biological explanation (8). Furthermore, the frequently observed adverse relationship between stressful events and RA (9) pointed to the upregulation of PRL in response to stress (10) as a contributing factor. However, other evidence suggested the opposite. A large population cohort, controlled for breastfeeding among parous women, did not support nulliparity as risk factor and revealed that breastfeeding for >12 months was inversely related to the development of RA (11). Also, RA improves or goes into remission during pregnancy (12) when the circulating levels of PRL and placental lactogen are high. Moreover, stress worsens but also attenuates RA dependent on the duration and type of stressors and in association with stress hormones, including PRL (9). For example, acute exposure to hyperprolactinemia enhances inflammation during stress, whereas chronic hyperprolactinemia is immunosuppressive (13). Finally, controversies were found when measuring PRL levels in the circulation of patients with RA (reviewed by Clapp et al., 2016 (2)). Higher, similar, and even lower PRL levels, within the normal range (≤ 20 μg/L), occurred with no clear association to disease severity. Lowering or increasing circulating PRL levels with dopamine D2 receptor agonists or antagonists, respectively, were both effective and ineffective against RA. Altogether the contrasting findings have indicated dual outcomes of PRL in RA and encouraged the search for clarifying mechanisms.

Opposite effects of PRL on the immune response have been known for more than three decades and are essentially associated to PRL concentration (14), with lower levels (≤25 μg/L) being immunostimulatory and higher levels (≤100 μg/L) immunosuppressive (15). It is possible that systemic levels of PRL in RA are confounded by PRL produced and metabolized at the inflamed joint. Infiltrated leukocytes and fibroblasts of the RA synovium produce PRL (16) and matrix metalloproteases (MMPs) upregulated in the joints of patients with RA (17) cleave this hormone to vasoinhibin (18), a PRL fragment with potent anti-angiogenic and pro-inflammatory properties (19). Following is a summary of the PRL/vasoinhibin axis, an integrative framework able to alter joint inflammation by influencing the levels of systemic and local PRL and vasoinhibin.

## The PRL/Vasoinhibin Axis

The PRL/vasoinhibin axis is a newly described endocrine axis where the proteolytic cleavage of PRL to vasoinhibin is regulated at the hypothalamus, the pituitary, and the target tissue levels (20). Disruption of this axis contributes to the pathogenesis and progression of diabetic retinopathy (21), retinopathy of prematurity (22), peripartum cardiomyopathy (23), pre-eclampsia (24), and inflammatory arthritis (25, 26). Vasoinhibin comprises a family of PRL fragments that range from 5.6 to 18 kDa that correspond to the first 48 to 159 amino acid residues of PRL depending on the cleavage site of proteases that include MMPs (18), cathepsin D (27), bone morphogenetic protein 1 (28), thrombin (29), and plasmin (30). Vasoinhibin signals through receptor/binding protein complexes distinct from the PRL receptor (31) to exert effects frequently opposite to those of the full-length hormone. PRL stimulates angiogenesis, whereas vasoinhibin inhibits angiogenesis, vasodilation, and vasopermeability (19). Vasoinhibin acts as proinflammatory cytokine upregulating inducible nitric oxide synthase (iNOS) in lung tissues (fibroblasts and type II epithelial cells) (32), whereas PRL attenuates proinflammatory cytokine-induced iNOS expression in these cells (33). PRL inhibits and vasoinhibin stimulates anxiety- and depression-related behaviors (34) as well as neuronal apoptosis (35), respectively. However, both PRL and vasoinhibin stimulate the release of vasopressin by the hypothalamo-neurohypophyseal system (36). Because opposing actions reside within the PRL molecule, proteolytic cleavage represents an efficient mechanism for balancing functions. Recent work showed that a short linear motif of just three residues (His46-Gly47-Arg48) is the functional antiangiogenic determinant of vasoinhibin and that such motif is concealed in PRL by salt-bridges between Arg48 and Glu161 and 162 located in PRL fourth alfa-helix, a part of PRL removed during vasoinhibin generation (37).

The influence of the PRL/vasoinhibin axis in arthritis is suggested by the presence of PRL in the synovial fluid and of PRL, vasoinhibin, and PRL-cleaving MMPs in joint tissues including chondrocytes, vascular endothelial cells, synoviocytes, fibroblasts, and immune cells (reviewed by Clapp et al., 2016 (2)). While RA remains a uniquely human disease, animal models with induced synovial inflammation are an essential component of drug development (38) that have helped investigate the influence of the PRL/vasoinhibin axis in RA.

## PRL, Vasoinhibin, and Induced Arthritis in Rodents

Murine adjuvant arthritis (AA) and antigen-induced arthritis (AIA) are models of inflammatory arthritis, including RA, where disease is mediated by antigen-specific immune responses by T and B lymphocytes (39). The AA model is commonly induced by a single intradermal injection of complete Freund’s adjuvant in rats and mice and is characterized by a reliable, rapid onset and progression of robust and easily measurable polyarticular inflammation, cartilage degradation, and bone loss. AIA is usually induced in mice immunized by intradermal and subsequent intra-articular injection of antigen (methylated bovine serum albumin) that causes acute monoarticular inflammation and eventual joint destruction.

A first study, carried out 40 years ago, suggested a detrimental effect of PRL by showing that hypophysectomized rats do not develop AA unless treated with this hormone (40). However, adrenocortical deficiency due to hypophysectomy confounded PRL action. In the absence of hypophysectomy, rats made hyperprolactinemic by placing anterior pituitary grafts under the kidney capsule showed less severe AA and higher corticosterone circulating levels (41). The PRL beneficial action was recently confirmed and extended by showing that hyperprolactinemia induced by anterior pituitary grafts, osmotic minipumps delivering PRL, or treatment with the dopamine D2 receptor blocker, haloperidol, reduced joint inflammation and pain, cartilage loss, and bone erosion in AA rats (42, 43). Reduced inflammation involved systemic (lower levels of circulating C-reactive protein and TNFα) and local mechanisms (43). The long isoform of the PRL receptor was upregulated in arthritic joints where hyperprolactinemia inhibited enhanced expression of proinflammatory cytokines (TNFα, IL-1β, IL-6, INFγ), elevated chondrocyte apoptosis, and increased osteoclast differentiation (42, 43). Furthermore, PRL-receptor null mice (*Prlr-/-)* exhibited a more severe AA (43), which was consistent with previous reports showing that targeted disruption of the PRL receptor (44) or PRL (45) enhances immune responses and mortality under stress-related conditions.

The positive role of PRL in murine arthritis contrasts with its controversial action in RA. Because high PRL levels are immunosuppressive (15), the magnitude of the induced hyperprolactinemia (>60 μg/L) (42, 43) could be an explanatory mechanism. Another contributing factor may be the cleavage of PRL to vasoinhibin. Hyperprolactinemia promotes the conversion of PRL to vasoinhibin by providing more substrate to cleaving proteases. Hyperprolactinemic mice overexpressing PRL in the liver have enhanced levels of circulating vasoinhibin (46), and pharmacologically induced hyperprolactinemia results in higher levels of vasoinhibin in ocular tissues and fluids of rats (47) and humans (48). In agreement, the activity of major PRL-cleaving proteases, MMPs and cathepsin D, is upregulated in the joints from AIA mice (26), vasoinhibin increases in the circulation of *Prlr-/-* mice when subjected to AIA (26), and *Prlr-/-* mice are hyperprolactinemic (49).

Upregulation of vasoinhibin can contribute to the beneficial outcome of PRL in arthritis by means of its inhibitory effects on blood vessels. Exacerbated vasopermeability and angiogenesis promote synovial inflammation and inhibition of angiogenesis is a promising therapy in RA (50). Hypervasopermeability results in edema formation and joint swelling, and pannus formation requires new blood vessels to cope with the increased requirement of oxygen and nutrients and the delivery of inflammatory cells and molecules. Consistent with this notion, the intra-articular delivery of the vasoinhibin gene *via* a recombinant adeno-associated type 2 vector (AAV2-Vi) reduced pannus vasopermeability and angiogenesis, joint inflammation, and bone loss in mice under severe AIA (25).

Nevertheless, the role of vasoinhibin in arthritis is not a simple matter, as vasoinhibin is also proinflammatory. Higher circulating vasoinhibin levels coincide with exacerbated arthritis in *Prlr-/-* mice (26, 43) and vasoinhibin has proinflammatory effects in lung tissues (32). A recent study showed that vasoinhibin indirectly inhibits and directly stimulates joint inflammation depending on vasoinhibin concentration and the severity of the disease in which it acts (26). While the AAV2-Vi vector indirectly (via an antiangiogenic mechanism) ameliorated severe joint inflammation (25), it enhanced arthritis under mild inflammatory conditions (26). Vasoinhibin gene delivery in mice subjected to mild AIA enhanced joint swelling, synovial leukocyte infiltration, and expression of proinflammatory mediators (*Il1b, Il6, Inos, Mmp3, Icam1, Cxcl1, Cxcl2, Cxcl3, and Ccl2*) by a direct action on synovial fibroblasts (26). The magnitude of vasoinhibin transgene expression was higher under mild vs. severe AIA suggesting that, depending on the inflammatory context, higher levels of vasoinhibin are needed to promote inflammation but not anti-inflammation in arthritis.

Altogether evidence shows that dual actions of PRL extend to vasoinhibin and are dependent on the local concentration of each hormone, the level of inflammation, the activity of local proteases, and the activation of specific cells and signaling pathways.

## Targeted Cells and Signaling Pathways

**Figure 1:**
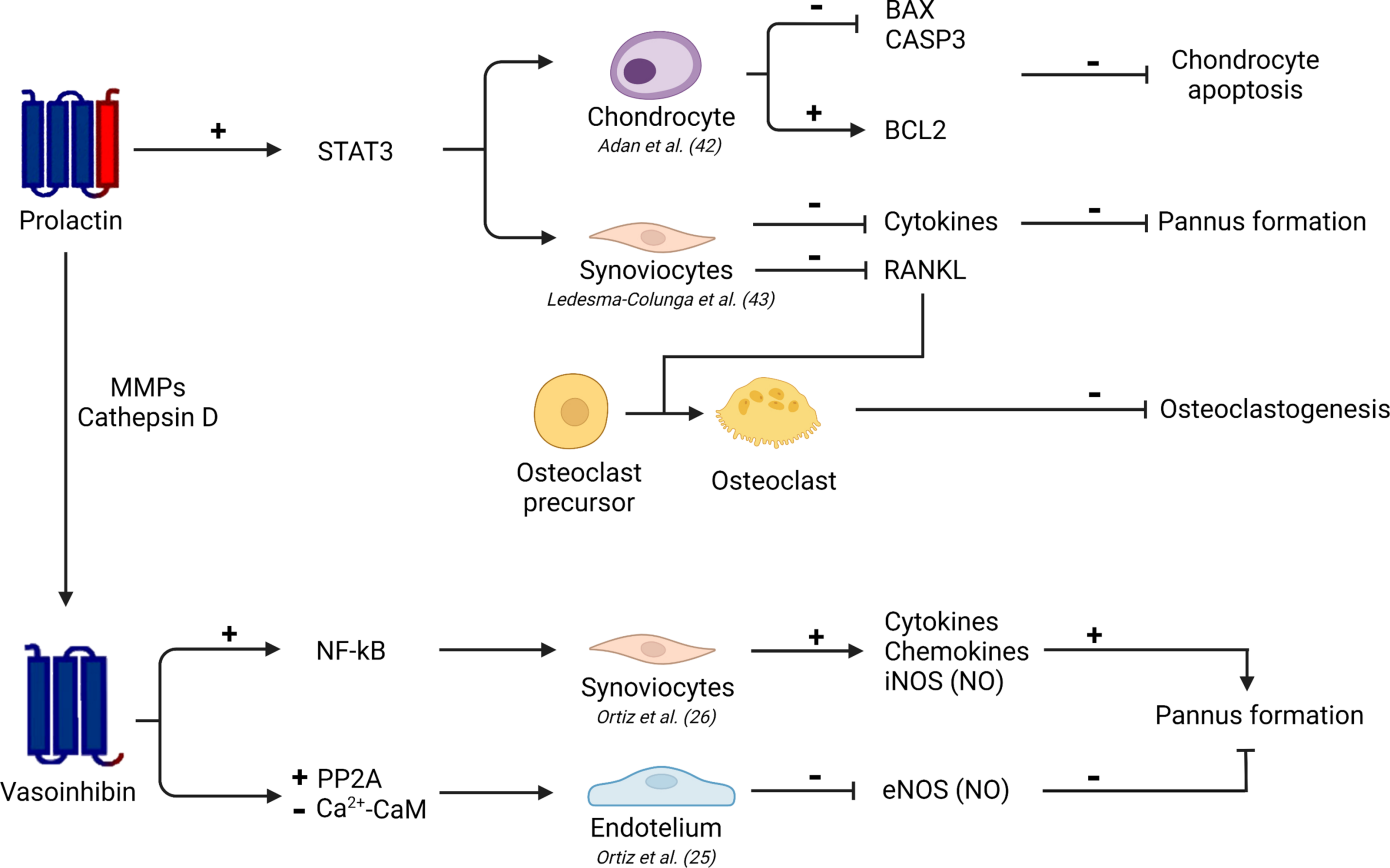
Schematic representation of PRL and vasoinhibin signaling in various cells of the joint. MMPs, matrix metalloproteases; STAT3, signal transduction activator of transcription 3; NF-kB, nuclear factor kappa-B; PP2A, protein phosphatase 2A; Ca^2+^- CaM, calcium-calmodulin complex; BAX, BCL2 associated X-protein; Bcl-2, B-cell lymphoma 2; CASP3, caspase 3; RANKL, receptor activator of nuclear factor κB ligand; iNOS, inducible nitric oxide synthase; NO, nitric oxide; eNOS, endothelial nitric oxide synthase. Scheme created with Biorender.com.

PRL signals directly on chondrocytes and synovial fibroblasts to inhibit cartilage degradation, synovial inflammation, and osteoclastogenesis in arthritis (42, 43). Articular chondrocytes express the long form of the PRL receptor (51) and PRL inhibits the apoptosis of cultured chondrocytes in response to proinflammatory cytokines (Cyt: IL-1β, TNFα and INFγ) by preventing the induction of p53 and decreasing the BAX/BCL-2 ratio through a NO-independent, JAK2-STAT3 dependent pathway (42) (**Figure 1**). Furthermore, the Cyt upregulate the long PRL receptor in synovial fibroblasts (43) which are key cells in the initiation and perpetuation of joint inflammation and destruction (52). PRL induces the phosphorylation/activation of STAT3 in cultured in synovial fibroblasts to inhibit Cyt-induced expression of IL-1β, IL-6, and receptor activator of nuclear factor κB ligand (RANKL), a major promoter of osteoclastogenesis in RA (43) (**Figure 1**).

In contrast to PRL, vasoinhibin acts on synovial fibroblasts to promote inflammation. Vasoinhibin activates the NFκB signaling pathway in cultured synovial fibroblasts to upregulate proinflammatory mediators, chemokines, and iNOS-mediated NO production (26) (**Figure 1**). However, like PRL, vasoinhibin inhibits inflammation albeit through the inhibition of vascular endothelial cells. Vasoinhibin signals on synovial endothelial cells to stimulate protein phosphatase 2A and inhibit the Ca^2+^-calmodulin binding that leads to blockage of the VEGF-induced endothelial NOS (eNOS) activation required for pannus vasopermeability and angiogenesis (25, 53) (**Figure 1**).

**Figure 2:**
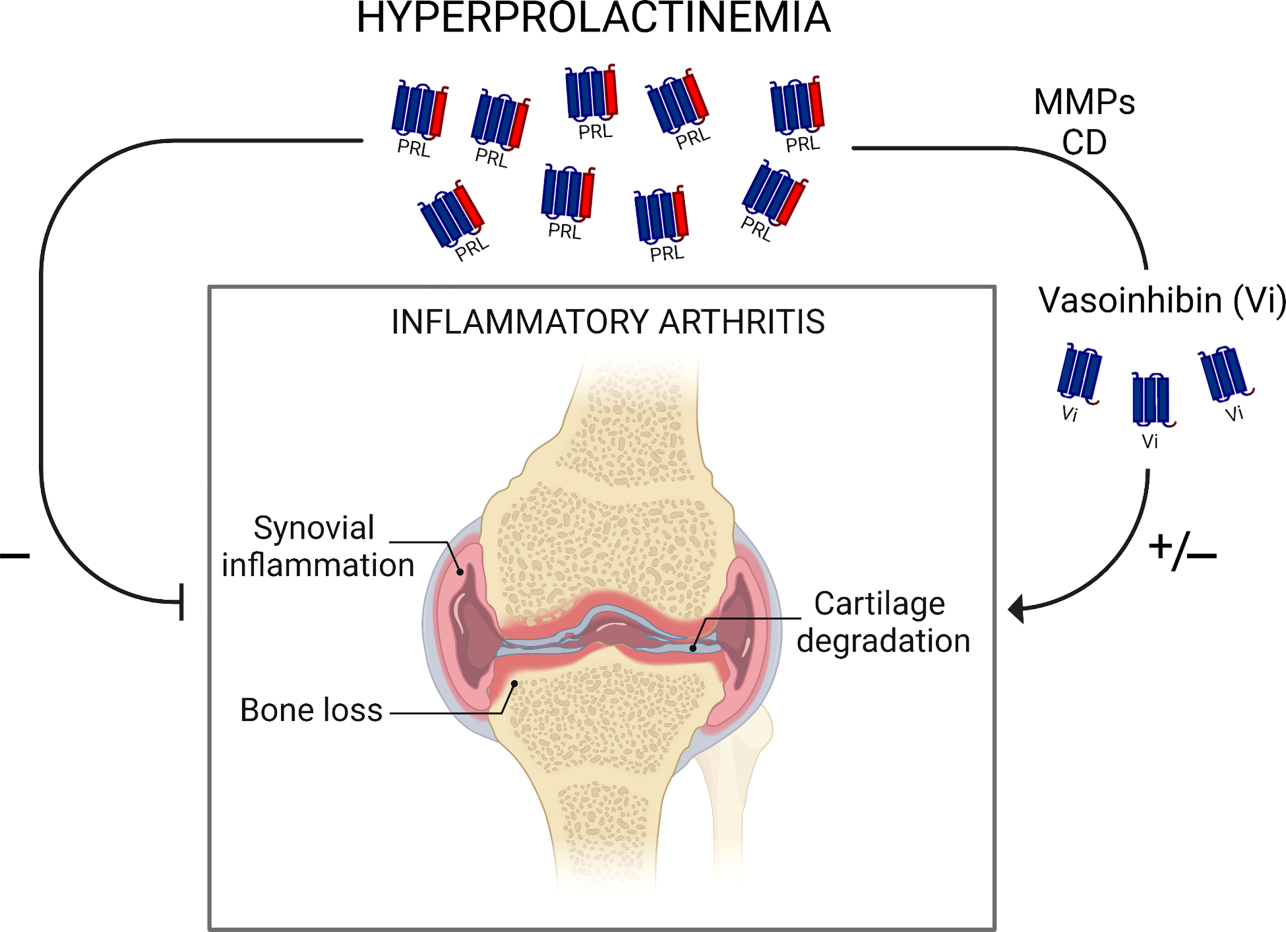
Schematic representation of findings in rodent models showing dual actions of hyperprolactinemia in inflammatory arthritis. Hyperprolactinemia inhibits synovial inflammation, cartilage degradation, and bone loss directly or *via* its proteolytic cleavage by matrix metalloproteases (MMPs) and cathepsin D (CD) to antiangiogenic vasoinhibin. PRL conversion to vasoinhibin may also worsen inflammatory arthritis by a vasoinhibin pro-inflammatory effect. Understanding how and when PRL and vasoinhibin actions operate and mechanistically interact to influence arthritis progression warrants further research. Scheme created with Biorender.com.

Dual actions on inflammation illustrate the complex balance of the inflammatory response. As PRL and vasoinhibin, major proinflammatory cytokines (INFγ, IL-2, IL-6, TNFα) function as anti-inflammatory mediators and classical anti-inflammatory factors (IL-10, TGFα, glucocorticoids) exhibit proinflammatory effects depending on cytokine concentration, the stage of the disease, and the combination with other cytokines (54, 55). Major questions are how and when PRL and vasoinhibin opposing actions operate and mechanistically interact to influence arthritis progression (**Figure 2**). Current data suggest that vasoinhibin generation is dependent on hyperprolactinemia and that the proinflammatory action of vasoinhibin on synovial fibroblasts may occur during the early mild phase of arthritis, whereas the anti-inflammatory effect, *via* inhibition of synovial vascular cells, manifest at a later, more severe stage (26). We hypothesize that these opposing actions work in concert to prevent infection and limit destruction of joint tissues.

## Clinical Implication

Animal models of RA are of limited therapeutic information since none of these models are truly RA. However, murine inflammatory arthritis has been extensively used for drug development (38) and has provided insights into the influence of the PRL/vasoinhibin axis on joint inflammation. Experimental studies showed that increasing prolactinemia, either by PRL infusion or treatment with the dopamine D2 receptor blocker haloperidol, ameliorates the severity of arthritis, either directly (42) or *via* the PRL conversion to antiangiogenic vasoinhibin (25) (**Figure 2**). Of note, a pilot clinical trial carried out 40 years ago showed that haloperidol improved the evolution of RA (56) and a recent observational study described the potential inverse association between haloperidol and RA (57). Also, inhibition of angiogenesis is a promising therapy for RA (50) and vasoinhibin itself may represent a therapeutic opportunity by virtue of its antiangiogenic and anti-vasopermeability properties. Translation of vasoinhibin into the clinic has been hampered by difficulties in its recombinant production (58). However, the barrier of using vasoinhibin as therapeutic agent was recently removed by showing that seven amino acid peptides containing the anti-angiogenic motif (HGR) of vasoinhibin inhibit angiogenesis and vasopermeability with the same potency as the whole protein (37). Oligopeptide optimization to target vascular and not non-vascular actions in arthritis represents a promising therapeutic approach and an important tool for guiding future research. Nonetheless, dichotomous actions of the PRL/vasoinhibin axis expose its intricate role in inflammatory arthritis and demand further research to better understand its role and therapeutic application in RA.

## Conclusions

The role of PRL in RA remains poorly defined but hyperprolactinemia is emerging as a protective influence. Evidence supporting the beneficial impact of physiological hypeprolactinemia (in pregnancy and after breastfeeding) on RA is reinforced by experimental studies showing that sustained PRL administration or genetic deletion of the PRL receptor ameliorates or worsens the severity of inflammatory arthritis, respectively. PRL signals on arthritic joint tissues (chondrocytes and synovial fibroblasts) to inhibit cartilage degradation, synovial inflammation, and osteoclastogenesis. Hyperprolactinemia promotes the conversion of PRL to vasoinhibin, a PRL fragment that directly stimulates and indirectly inhibits (via an antiangiogenic mechanism) joint inflammation in a context- and cell type-dependent manner. Understanding the mechanisms governing the regulation and action of the PRL/vasoinhibin axis in inflammatory arthritis should help clarify the role of PRL in RA to ultimately develop novel therapeutic interventions that can be tested in patients.

## Author Contributions

CC wrote the manuscript. GO, JG-R, ML-C, FM-D, NA, and GME edited, and revised the manuscript. All authors contributed to the article and approved the submitted version.

## Funding

This work was supported by grants from UNAM DGAPA-PAPIIT IN202321 and L’Oréal-UNESCO-AMC-CONALMEX to CC.

## Conflict of Interest

The authors declare that the research was conducted in the absence of any commercial or financial relationships that could be construed as a potential conflict of interest.

## Publisher’s Note

All claims expressed in this article are solely those of the authors and do not necessarily represent those of their affiliated organizations, or those of the publisher, the editors and the reviewers. Any product that may be evaluated in this article, or claim that may be made by its manufacturer, is not guaranteed or endorsed by the publisher.
